# Phase II trial of docetaxel, cisplatin and fluorouracil followed by carboplatin and radiotherapy in locally advanced oesophageal cancer

**DOI:** 10.1038/sj.bjc.6603585

**Published:** 2007-01-23

**Authors:** V Chiarion-Sileni, L Corti, A Ruol, R Innocente, C Boso, P Del Bianco, J Pigozzo, R Mazzarotto, O Tomassi, E Ancona

**Affiliations:** 1 Medical Oncology Unit, Istituto Oncologico Veneto, Padova, Italy; 2 Radiotherapy Unit, Istituto Oncologico Veneto, Padova, Italy; 3 Third Surgical Clinic, University Hospital, Padova, Italy; 4 Department of Radiotherapy, CRO Aviano, Italy; 5 Clinical Trials and Biostatistics Unit, Istituto Oncologico Veneto, Padova, Italy; 6 Medical Oncology Unit, General Hospital, Castelfranco Veneto, Italy

**Keywords:** chemoradiotherapy, docetaxel, oesophageal cancer, phase II study

## Abstract

This study was performed to assess the efficacy and safety of docetaxel, cisplatin and fluorouracil combination in patients with unresectable locally advanced oesophageal squamous cell carcinoma. Treatment consisted of docetaxel 60 mg m^−2^, cisplatin 75 mg m^−2^ on day 1 and fluorouracil 750 mg m^−2^ day^−1^ on days 2–5, repeated every 3 weeks for three cycles, followed by carboplatin 100 mg m^−2^ week^−1^ for 5 weeks and concurrent radiotherapy (45 Gy in 25 fractions, 5 days week^−1^). After radiotherapy, eligible patients either underwent an oesophagectomy or received high dose rate endoluminal brachytherapy (HDR-EBT). Thirty-one out of 37 enrolled patients completed the planned chemotherapy and 30 completed chemoradiation. After completion of chemotherapy, 49% (95% CI: 32.2–66.2) had a clinical response. Twelve patients (32%) underwent a resection, which was radical in 60% (postoperative mortality: 0%). A pathological complete response was documented in four patients (11% of enrolled, 30% of resected). The median survival was 10.8 months (95% CI: 8.1–12.4), and the 1- and 2-year survival rates were 35.1 and 18.9%, respectively. Grade 3–4 toxicities were neutropoenia 32%, anaemia 11%, non-neutropoenic infections 18%, diarrhoea 6% and oesophagitis 5%. Nine patients (24%) developed a tracheo-oesophageal fistula during treatment. Even if the addition of docetaxel to cisplatin and 5-fluorouracil (5-FU) seems to be more active than the cisplatin and 5-FU combination, an incremental improvement in survival is not seen, and the toxicity observed in this study population is of concern. In order to improve the prognosis of these patients, new drugs, combinations and strategies with a better therapeutic index need to be identified.

Oesophageal cancer is the sixth leading cause of death from cancer worldwide (Pisani *et al*, 1999) and its fatality rate at 5 years reaches 90% (Blot and McLaughlin, 1999). The prognosis remains dismal because, at diagnosis, surgery is inappropriate in 40–60% of patients owing to unresectable primary disease, distant metastases or high operative risk (Sagar *et al*, 1994).

Of those undergoing operation, between 55 and 80% will have a ‘potentially’ curative resection (Muller *et al*, 1990; Sagar *et al*, 1994), which is safe only in experienced hands (Siewert *et al*, 2001).

With radiotherapy alone these results did not improve, and 5-year survival rates remain <10% (al-Sarraf *et al*, 1997). Chemotherapy can provide significant palliation of symptoms for patients with unresectable, locally advanced or metastatic disease. The repertoire of chemotherapeutic agents with activity against squamous cell carcinoma (SCC) is restricted, but responses have been documented in studies utilising cisplatin, 5-fluorouracil (5-FU), vindesine, mytomicin, paclitaxel and vinorelbine (Enzinger *et al*, 1999). In the multi-modality treatment of oesophageal cancer, cisplatin and continuous-infusion 5-FU (PF), alone or combined with radiotherapy, is the most frequently used regimen (Cooper *et al*, 1999).

The response rate reported with PF ranged from 35 to 40% (De Besi *et al*, 1986; Bleiberg *et al*, 1997; Ancona *et al*, 2001), whereas the 2-year survival rates of patients with locally advanced oesophageal cancer ranged from 8 to 55%, with a mean 27%. A better prognosis with chemoradiotherapy/chemotherapy (al-Sarraf *et al*, 1997; Stuschke *et al*, 2000; Kodaira *et al*, 2003; Polee *et al*, 2003) has been reported, but these series also included patients with potentially resectable carcinomas.

The majority of studies on combined therapy showed longer survival for patients achieving a pathologic complete response (pCR), which can be obtained in 12–40% of patients treated with PF combined therapy (Forastiere *et al*, 1997; Ancona *et al*, 2001). Patients with a less advanced stage are more likely to achieve a pCR than those with a more advanced disease (Rohatgi *et al*, 2005).

Therefore, there is a need to test new combinations, specifically in unresectable locally advanced oesophageal patients, with the aim of increasing the pCR rate and survival.

The mechanism of action of docetaxel is different from that of PF and has proved to have an additive effect with cisplatin and supra-additive antitumour activity with fluorouracil *in vitro* and in murine models of xeno-grafted human tumours (Burris *et al*, 1992; Catimel *et al*, 1994; Dreyfuss *et al*, 1996). Docetaxel has been shown to be more active than paclitaxel in 10 oesophageal cancer cell lines (Kawamura *et al*, 1997) and clinically active in patients with oesophageal adenocarcinoma (Einzig *et al*, 1996). Furthermore, its combination with cisplatin, fluorouracil and leucovorin showed an overall response (OR) of 94% and a complete response (CR) of 44% in patients with SCC of the head and neck, without altering the ability to administer definitive radiotherapy (Colevas *et al*, 1998).

It seemed therefore appropriate to evaluate, in a phase II study, the combination of docetaxel, cisplatin and fluorouracil (DCF) in patients with locally advanced oesophageal SCC.

Italian patients with oesophageal SCC are heavy smokers and drinkers, and often present vascular and peripheral nerve impairment from this abuse. Therefore, we chose to use carboplatin instead of cisplatin during radiotherapy, as continuous carboplatin infusion during radiotherapy has been shown to be cytotoxic as cisplatin in human lung cancer cell lines (Groen *et al*, 1995a), and active, while less nephro- and neurotoxic, in non-small-cell lung and head and neck cancer patients (Groen *et al*, 1995b; Loreggian *et al*, 1997).

The study reported here is a prospective phase II trial of DCF, followed by external beam radiotherapy concurrent with continuous carboplatin infusion, designed to evaluate the activity of DCF in patients with locally advanced oesophageal SCC.

Secondary objectives were safety of the combination, the pCR rate in operable patients, local and systemic recurrence and median and overall survival.

## PATIENTS AND METHODS

### Eligibility and pretreatment evaluation

Before entry into the study, all patients were evaluated by a multidisciplinary team, which included a medical oncologist, a radiation therapist, a surgeon and a gastroenterologist. Eligibility criteria were age <75 years, histologically proven and previously untreated SCC of the oesophagus, WHO performance status (PS) ⩽2, absolute neutrophil count ⩾2 × 10^9^ l^−1^, platelet count ⩾100 × 10^9^ l^−1^, and adequate renal and hepatic function. Exclusion criteria were evidence of distant metastases, pleural or pericardial effusion, fistulisation, prior malignancies (other than basal cell skin carcinoma), prior myocardial infarction or uncontrolled infections. All patients were required to give written informed consent before entering the study, which was approved by the Ethics Committee of the two participating centres.

Pretreatment evaluation included a medical history, physical examination (PE), complete blood cell count and serum chemistry tests; barium oesophagram; oesophagoscopy; bronchoscopy; endoscopic ultrasound (EUS); computed tomography (CT) scans of the chest and abdomen; cervical US with fine-needle aspiration biopsy of suspicious nodes.

Based on the results of the EUS, patients were assigned a preoperative clinical stage according to the 1997 TNM classification of the International Union Against Cancer (Sobin, 1997). In case of a discrepancy between the CT and EUS findings, we classified the patient according to the worst stage. Only those patients with T3–4 or N1 or M1 (nodal) disease, unresectable for tracheobronchial infiltration, or an M1a nodal extension, or laryngeal nerve palsy were eligible for this study. One patient with a T2 N1 tumour was also considered eligible owing to the cervical location.

### Treatment plan

Treatment consisted of docetaxel 60 mg m^−2^, as a 1-h infusion, with 500 ml normal saline on day 1 of a 21-day cycle. Cisplatin 75 mg m^−2^ was given 1 h after the completion of docetaxel over 30 min with a 2 l fluid pre-hydration (0.9% saline+30 ml of 10% KCl+12 ml of 10% MgCl_2_), followed by a 1.5 l fluid post-hydration (0.9% saline+30 ml of 10% KCl+12 ml of 10% MgCl_2_). Fluorouracil 750 mg m^−2^ day^−1^ was given by intravenous (i.v.) continuous infusion on days 2–5.

Blood products, nutritional support and all other means of symptomatic relief were allowed according to each patient's needs. Granulocyte colony-stimulating factor, 300 *μ*g day^−1^ subcutaneously, was allowed only in case of neutropoenic fever.

Dose modifications were planned as follows: for haematological toxicity consisting of absolute neutrophil count (ANC) less than 1500 cells mm^−3^ or platelet count less than 100 000 cells mm^−3^ on day 1, delay of DCF for 1 week; reduction of the dose of all drugs to level –1 (−25%) for ANC <500 cells mm^−3^, without fever, or platelet count <50 000 cells mm^−3^ without bleeding, or grade 3 mucositis according to the National Cancer Institute common toxicity criteria (NCI-CTC, version 2.0), and to level −2 (−40%) for ANC <500 cells mm^−3^ with fever, or platelet count <50 000 cells mm^−3^, with bleeding, or grade 4 mucositis; reduction of cisplatin to level –1 for grade 2 renal toxicity and reduction to level –2 for grade 3 renal toxicity.

Interval assessments during the treatment included PE, nutrition evaluation, weekly complete blood cell count with differential and platelet count, and renal and liver function tests every 3 weeks.

Radiation therapy was administered with a high-energy linear accelerator at 6–20 mV. The treatment volume included the area of the primary tumour and potential sites of lymphatic involvement. The planned target volume for carcinoma of the upper or middle third oesophagus included the primary tumour with a 5-cm longitudinal margin, metastatic nodes with a 2-cm margin, supraclavicular fossa and mediastinum. For carcinoma of the lower third oesophagus, the field was extended to include both the peri-gastric and celiac nodes. The dose was prescribed to the isodose line, which covered the volume at risk. A daily dose of 1.8 Gy was given up to a dose of 30.6 Gy. The radiation portals were then changed to encompass the primary tumour and metastatic nodes with a 2-cm margin lesion up to a final dose of 45 Gy. The radiation dose to the spinal cord was maintained at a maximum of 45 Gy.

Carboplatin, 100 mg m^−2^ week^−1^ i.v. continuous infusion, was concurrently administered with radiotherapy for 5 weeks by an elastomeric infusion pump through a central venous access catheter. Radiation therapy and carboplatin were withheld only if the ANC was <500 or the platelet count <25 000.

Patients who were unable to maintain their weight were started on enteral or parenteral feeding. Patients were withdrawn from the study if they presented progressive disease, life-threatening toxicities, fistulisation or expressed their desire to withdraw.

### Surgery, brachytherapy and staging criteria

Toxicity was graded using the NCI-CTC version 2.0. All events that occurred during the treatment or worsened in comparison with baseline scores were considered toxic.

Patients were restaged 4 weeks after the third DCF cycle and 4–6 weeks after completion of chemoradiation, repeating the pretreatment staging work-up. Clinical CR was defined as a complete symptomatic, radiographic and endoscopic normalisation.

Definition of a clinical partial response (PR) was more difficult; we defined PR as a reduction from the initial clinical stage (AJCC, 1997) (Fleming, 1997), determined by a compilation of results from the CT scans, EUS, barium oesophagram and endoscopy.

Stable disease (SD) was defined as no change in the TNM stage, and progressive disease (PD) as an increase in the TNM stage or by the discovery of new metastatic lesions at any time. Patterns of failure were defined as the first site of failure. Local failure included the primary tumour and local/regional lymph nodes. Distant failure included any other site of disease recurrence. Dysphagia was scored before the treatment and at each subsequent PE, using a published dysphagia scale (Knyrim *et al*, 1993): a score of 0 denoted no dysphagia; 1, trouble in swallowing solids; 2, the ability to swallow semi-solids alone; 3, the ability to swallow liquids alone; and 4, complete dysphagia to solids and liquids.

Patients who obtained a CR or a PR were offered an Ivor-Lewis oesophagectomy. Resection and reconstruction were accomplished using simultaneous right thoracotomy and midline laparotomy. For cervical and upper thoracic lesions, a concomitant pharyngolaryngectomy was carried out together with a three-field lymphadenectomy. Pathological complete remission (pCR) was defined as the absence of residual tumour in the resected oesophageal and nodal specimen (pT0N0). Patients who refused or were considered unfit for surgery were offered a high dose rate endoluminal brachytherapy (HDR-EBT) boost with the aim of improving local control. High dose rate endoluminal brachytherapy was performed once weekly for 3 consecutive weeks and started 2–4 weeks after chemotherapy and radiotherapy completion, to allow resolution qof oesophageal mucositis. A radio-opaque clip was placed on the oesophageal mucosa, 1 cm above the tumour bed, via endoscopy. This device substantially facilitates the radiological localisation of the tumour bed when positioning the applicator charged with a dummy source. The mean total dose was 15 Gy, 5 Gy per session, prescribed at 0.6 cm from the surface of a flexible applicator with a diameter of 0.8 cm. The active length, planned at the time of endoscopy, was the visible mucosal tumour with a 2 cm distal and proximal margin, without optimisation.

### Statistical methods

The primary outcome of this study was the OR rate, defined as the proportion of responders (complete or partial) among all patients, of the DCF combination in locally advanced oesophageal SCC patients. The sample size was calculated based on the assumption that a 40% OR rate or less was considered insufficient to warrant further investigation. On the other hand, a probability of response above 60% would be clinically sufficient and indicates that further investigation of this regimen is appropriate. The accrual consisted of two stages according to Simon (1989).

This design minimises the expected number of treated patients, if the response rate is inadequate. If seven or less responses were observed in the first 19 patients, the study would have been terminated. In the case of eight or more responses in these 19 patients, 15 additional patients would have been enrolled. If more than 16 responses were observed among all of the 34 patients, the treatment would be considered active. This scheme ensured that the chance of erroneously rejecting the treatment is less than 10% if the treatment is active in at least 60% of the patients, whereas the probability of erroneously recommending a treatment whose response rate is inadequate is limited to 15%. Confidence intervals were adjusted for the two-stage design (Atkinson and Brown, 1985). Secondary end points were the pathological remission rate, the toxicity rate, dysphagia response and survival times.

Survival times were calculated from the beginning of chemotherapy to the date of death, progressive disease or to last follow-up. The Kaplan–Meier method was used to estimate the survival curves.

Actual dose intensity was calculated as recommended by Hryniuk and Goodyear (1990).

Analyses were performed using the SAS statistical package (SAS, release 8.02, Cary, NC, USA)

## RESULTS

### Patient characteristics

Between July 1998 and February 2002, 37 patients were enrolled in the study at two Italian oncology centres: the Medical Oncology Unit of the Istituto Oncologico Veneto, Padova and the Centro di Riferimento Oncologico, Aviano. Characteristics of the 37 patients are listed in Table 1. The median age was 61 years (range, 39 – 72 years), and 62% of patients had a WHO PS of 2.

Twenty-six patients had T4 disease, 31 had mediastinal, two coeliac and three cervical node metastases; one patient had unsuspected bone metastasis. Twenty-five primary tumours were in the upper–mid-thoracic oesophagus, four in the cervical, two in the lower thoracic and six patients had more than one location. All patients had dysphagia before treatment: four (11%) grade 1; 16 (43%) grade 2; 11 (30%) grade 3; six (16%) grade 4 and almost 50% of patients had suffered a weight loss ⩾10%. According to Simon's design, 19 patients entered the first stage of study . Fifteen patients were required for the second stage. As it was expected that some patients might not be fully evaluable, 37 patients were finally recruited.

### Response and dysphagia relief

Response results are summarised in Table 2. Among the first 19 patients, 10 responses were observed. The OR rate, on an intention-to-treat analysis, was 48.6% (18 of 37 patients) (95% adjusted CI: 32.2 – 66.2%). Six patients (16%) had a CR with negative biopsies, 12 (32%) obtained a PR, seven patients (19%) remained stable, eight (22%) had PD and four (11%) early death. After carboplatin infusion and radiotherapy, two patients had a further reduction of disease, 20 remained stable and eight had progressive disease. All patients reported some grade of dysphagia on trial entry. Median baseline score was 2 (range 1–4). After induction chemotherapy, dysphagia improved by at least one level in eight patients (22%) and completely resolved in 12 patients (32%), with an overall improvement in dysphagia in 54% of patients. Dysphagia worsened in eight (22%) and six required placement of an enteral feeding tube for nutritional support. Nine (24%) developed a tracheo-oesophageal fistula, four during induction therapy and five during or at the end of radiotherapy.

### Surgery and brachytherapy

Of the 12 patients who underwent surgery, seven (58%) had a complete surgical resection with clear margins, two had a macroscopic clearance but positive resection margins on histological assessment, three had a macroscopically incomplete resection owing to tumour adherence to the trachea in two and to the aorta wall in one. There were no postoperative deaths and a pCR was documented in four patients (30% of resected and 10.8% of enrolled, respectively).

All the seven patients treated with HDR-EBT recurred locally.

### Toxicity and treatment delivery

Toxicity data are available for all patients. The most frequently encountered toxicities were leucopoenia (38% grade III/IV), neutropoenia (32% grade III/IV), anaemia (11% grade III/IV), mucositis (35% grade II and 5% grade III), non-neutropoenic infections (13% grade III/IV) and diarrhoea (6% grade III/IV) (Table 3). All patients experienced grade II alopecia. One patient died of a cerebral stroke (3% grade IV). Among the non-neutropoenic infections, we observed two, non-fatal, mycotic pneumonitis. Two fatal mediastinitis, due to a fistulisation, occurred immediately after the first cycle. One patient died after the third cycle owing to a neutropoenic fever and diarrhoea.

The cycles delivered were 103 over 111 planned; delays in chemotherapy owing to toxicity were required in three patients, with a maximum delay of 14 days. No dose reductions were made.

The planned dose intensity was 20 mg m^−2^ week^−1^ for docetaxel, 25 mg m^−2^ week^−1^ for cisplatin and 750 mg m^−2^ week^−1^ for fluorouracil.

The median percentage of dose intensity delivered was 96% for docetaxel, 98% for cisplatin and 98% for fluorouracil (Table 4).

The main toxicity during chemoradiotherapy was grade III and IV oesophagitis in 73% of patients. All patients received the planned weekly dose of carboplatin without delay and/or evidence of haematological toxicity. Thirty patients received the carboplatine-radiotherapy segment with a mean total radiation dose of 45 Gy (range 30–60 Gy). One patient stopped XRT after 30 Gy owing to the occurrence of a tracheo-oesophageal fistula and two patients continued to 60 Gy because they were unfit for surgery.

After carboplatin and radiotherapy, 18 downstaged patients were referred to the surgeon: 12 were operated, one refused and the remaining five were considered medically unfit for surgical resection. These six patients and one with a cervical tumour location were treated with HDR-EBT.

Two patients died of a myocardial failure 225 and 479 days from the start of therapy, one of them without evidence of disease.

### Survival and pattern of failure

**Figure 1:**
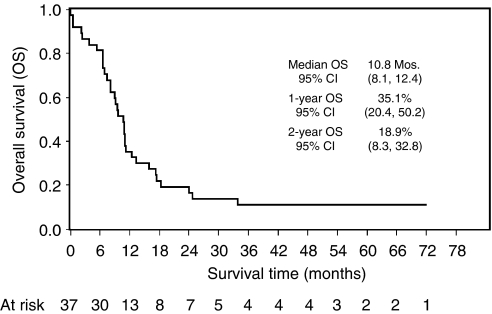
Overall survival for all patients. Survival time was calculated from the start of chemotherapy to the date of death or to the last follow-up.

**Figure 2:**
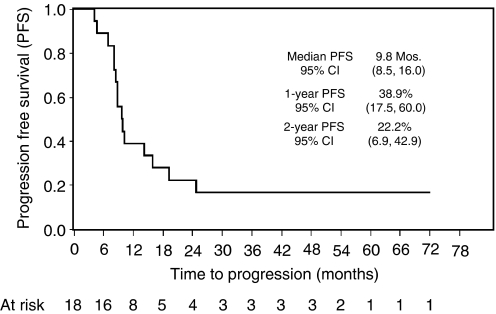
Progression-free survival for responding patients. Time to progression was calculated from the start of chemotherapy to the date of progression or to the last follow-up.

The median survival was 10.8 months (95% CI: 8.1–12.4), and 1- and 2-year survival rates were 35.1% (95% CI: 20.4–50.2) and 18.9% (95% CI: 8.3–32.8) (Figure 1). Currently, of 37 DCF-treated patients, four (11%) are alive and disease-free, with a minimum follow-up of 50 months. Fifteen out of the 18 responding patients progressed or relapsed. The median time to progression was 9.8 months (95% CI: 8.5 – 16.0) and 1- and 2-year progression-free survival rates were 38.9% (95% CI: 17.5 – 60.0) and 22.2% (95% CI: 6.9 – 42.9), respectively (Figure 2). The median survival of DCF responding patients was 14.7 months (95% CI: 11 – 24.7) *vs* 6.6 months (95% CI: 3.9 – 9.8) of non-responding.

Thirty patients are evaluable for progression site: 10 patients (33.3%) had a local progression, four (13.3%) had distant metastases without local recurrence and 16 (53.3%) had both. The most frequent sites of distant metastases were lungs (11 patients), bone (five), cutis and subcutis (three).

## DISCUSSION

In this population of locally advanced oesophageal SCC patients, DCF showed substantial activity in the treatment of oesophageal cancer. The 49% response rate observed is higher than that reported with cisplatin and 5-FU alone (De Besi *et al*, 1986; Bleiberg *et al*, 1997), which is still considered the standard treatment.

In the last decade, several attempts have been made to test new agents, especially taxanes, topoisomerase I inhibitors and vinorelbine. However, differences in the study population make the comparison difficult, as some studies had included both locally advanced tumours and metastatic disease, or metastatic disease alone, and others had included both adenocarcinoma and SCC. Despite a similar chemosensitivity reported for adenocarcinoma and SCC (Ilson *et al*, 1997), the adenocarcinoma tumour type was an independent prognostic parameter in a large Western world series (Siewert *et al*, 2001).

Paclitaxel used alone at different doses or combined with cisplatin gave an RR of 14.5–50% (Ajani *et al*, 1994; Kelsen *et al*, 1997; Petrasch *et al*, 1998) with a treatment-related mortality of 10% and hospitalisation owing to toxicity in 50% of the patients.

Irinotecan was evaluated in combination with cisplatin in a weekly schedule, reporting 20 responses in 35 patients (57%) in the first publication, although in a subsequent publication from the same author, only 10 (36%) confirmed major responses were reported (Ilson *et al*, 1999; Ilson, 2004).

Irinotecan was also combined with docetaxel, obtaining an RR of 30 %, in metastatic patients, with an extremely high incidence of febrile neutropoenia (43%) (Govindan *et al*, 2003).

The combination of vinorelbine and cisplatin was tested only in metastatic patients, obtaining an RR of 34% with acceptable toxicity (Conroy *et al*, 2002).

Docetaxel alone was tested in 52 metastatic patients, obtaining an RR of 20% with a median survival time of 8.1 months; febrile neutropoenia was the main toxicity and was observed in 18% of the patients (Muro *et al*, 2004). Docetaxel was also investigated in combination with cisplatin, obtaining four responses out of 10 patients with metastatic SCC (Laack *et al*, 2005) and in combination with capecitabine, obtaining 11 responses out of 24 patients with metastatic disease (Lorenzen *et al*, 2005).

The combination of docetaxel, cisplatin and 5-FU, evaluated in recurrent and previously treated patients, achieved major responses in four out of 10 patients; however, a grade 3/4 leukopoenia occurred in eight of them (Tanaka *et al*, 2003).

Our study is the first to test this combination in a homogenous population of previously untreated squamous oesophageal carcinoma patients, even if characterised by poor prognostic characteristics: 62% of patients had a grade 2 WHO PS, 84% had nodal involvement, 14% had non-regional lymph node metastases (M1a) and 89% had a primary lesion located above the carina. The median survival expected with palliative treatment as radiotherapy or intubation with self-expanding stents in these patients is only 3–6 months (Knyrim *et al*, 1993). The RR of 49% and the median survival of 10.8 months, with an improvement of dysphagia in more than 50% of patients, and a 1- and 2-year survival of 35 and 19%, clearly suggest that DCF combination is active, that chemoradiotherapy is the best palliation for patients with inoperable locally advanced oesophageal SCC and that this approach might be curative in some.

The treatment of these patients presents significant clinical problems related not only to the extension and location of disease, but also to their poor clinical and nutritional status owing to the long use of tobacco and alcohol, and made worse by the feeding impairment, resulting in an increase in complications, as well as toxic and early deaths. These complications are more frequent in SCC than in adenocarcinoma patients, as observed by others (Adelstein *et al*, 2000). In our study, the early deaths, partly related to disease complications and partly related to treatment toxicity, were 10.8%. This clearly disappointing percentage is inferior to the 18% reported by Adelstein *et al* (2000) with a paclitaxel combination, and has been previously reported in oesophageal cancer, even in patients with a less advanced stage of disease (Bleiberg *et al*, 1997; Wright *et al*, 1997). We observed that only patients with a WHO PS of 2 developed fatal toxicity; therefore, careful patient selection, improvement in liquid and nutritive intake, as well as the prophylactic use of antibiotics could be beneficial and recommendable before starting therapy. The high percentage of fistulisation observed might be explained by the prevalence of T4 lesions, localised mostly in the upper or middle portion of the oesophagus. The high risk of treatment-related perforation of the oesophageal wall in T4 oesophageal patients was also highlighted by Ohtsu *et al* (1999). In order to reduce the perforation risk, we decided to use induction chemotherapy, before chemoradiotherapy, with the hope of decreasing the tumour volume before encountering severe oesophagitis and stomatitis. Unfortunately, this strategy seemed to be ineffective, even if the dysphagia improved in the majority of patients.

The placement of a self-expanding feeding tube might have been more effective in preventing this complication; however, it was inapplicable in some patients owing to the extension or the location of the lesion, was not accepted by others and, moreover, might have interfered with the subsequent response evaluation. A change in the chemotherapy schedule, like the use of weekly doses, might help to reduce the acute tumour necrosis and permit wall repair (Ilson *et al*, 2003).

In this study, only five patients had M1 disease, making a comparison between them and other patients inadequate. However, it should be pointed out that three of them responded to the treatment, one obtained a pCR and another is one of the four long living. These findings are similar to the results reported by Ohtsu *et al* (1999)) and Polee *et al* (2003)) and support the concept that chemoradiotherapy has the same curative potential, for locally advanced disease, irrespective of mediastinal or M1a node extension; therefore, separating these groups is not strictly necessary. The utility of surgical resection after chemoradiotherapy in advanced oesophageal cancer is still not established; in spite of this, we offered responding patients the possibility of resection, as this was the best way to ascertain the rate of complete response, and residual tumour resection could be beneficial. Currently, only radically resected patients are long survivors in our study, despite evidence of residual tumour in three of them.

This result, together with that of others, seems to imply that surgical resection could be useful at least in some patients (Adelstein *et al*, 2000; Ilson *et al*, 2003). No conclusions can be drawn on the utility of HDR-EBT in the local control of disease from our data, as few patients received this treatment. We can, however, say that the planned dose of 15 Gy in three fractions was well tolerated with minimal acute effects, even if all patients progressed. Local recurrences (87%), all within the radiation field, remain the most important cause of death. We can argue that the dilation of radiotherapy and its combination with carboplatin alone could have hampered the adequate local control of the disease. Nevertheless, a similar rate (77%) of local failure was reported by Ohtsu *et al* (1999) who delivered a total dose of 60 Gy concomitant with PF, followed by two more courses of PF alone. Thus, this high rate of local recurrences is more likely related to the advanced stage of the disease than to the dilation of radiotherapy, or to the dose and schedule used.

Our results suggest that DCF is an active regimen with curative potential in some patients with unresectable locally advanced oesophageal SCC. However, survival is still disappointing and not improved over standard cisplatin/5-FU regimens. The toxicity observed in treating these patients is still relevant and in someway might hamper the clinical efficacy. In order to improve the prognosis of these patients, new drugs, combinations and strategies with a better therapeutic index need to be identified.

## Figures and Tables

**Table 1:** Patient characteristics

**Characteristics**	**No. of patients**	**%**
Total	37	
*Age (years)*		
Median (range)	61 (39–72)	
*Gender*		
Male	29	78
Female	8	22
*ECOG*^a^ *performance status*		
1	14	38
2	23	62
*Clinical stage*		
T2 N1	1	3
T3 N1	5	13
T4 N0–1	26^b^	70
T3–4 N0–1 M1a	5	14
*Tumour location*		
Cervical	4	11
Upper-middle	25	68
Lower	2	5
More than one site	6	16
*Weight loss >10%*	18	49

a ECOG=Eastern Cooperative Oncology Group.

b One of these patients had bone metastases.

**Table 2:** Response rate to chemotherapy

	**DCF^a^ response**		
**Responses**	**No. of patients**	**%**	**95% CI**
*Overall response*	18	49	32–66
*Complete response*	6		
*Partial response*	12		
Stable disease	7	19	
Progressive disease	8	22	
Early death	4	11	

a Docetaxel, cisplatin and fluorouracil.

CI=confidence interval.

**Table 3:** Docetaxel, cisplatin and fluorouracil chemotherapy toxicity

	**Common toxicity criteria (%) (no.=37)**			
**Toxicities**	**Grade 1**	**Grade 2**	**Grade 3**	**Grade 4**
Leucopoenia	39	19	27	11
Neutropoenia	29	19	24	8^a^
Thrombocytopoenia	8	8	5	0
Anaemia	46	22	8	3
Mucositis	27	35	5	0
Alopecia	0	100	0	0
Nausea and vomiting	19	13	3	0
Diarrhoea	0	0	3	3
Hepatic	3	0	0	0
Infection	0	0	3	8
Cerebrovascular	0	0	0	3^a^
Fistula	0	0	0	11^a^

a Toxic deaths occurred in four patients.

**Table 4:** Dose of docetaxel, cisplatin and fluorouracil and actual dose intensity

	**Docetaxel**	**Cisplatin**	**Fluorouracil**
	**Median (range)**	**Median (range)**	**Median (range)**
Target dose (mg m^−2^)	180	225	9000
mg m^−2^ received	176.5 (59.2; 218.2)	222.8 (68.2; 226.7)	8977.5 (2574.0; 9069.8)
Target DI,^a^ mg m^−2^ week^−1^	20	25	1000
Actual DI, mg m^−2^ week^−1^	19.3 (10.7; 24.0)	24.6 (3.3; 29.2)	984.4 (333.7; 1176.7)
% of target DI	0.96 (0.53; 1.20)	0.98 (0.13; 1.17)	0.98 (0.33; 1.18)

a DI=dose intensity.
